# Arthroscopy Assisted Reduction Percutaneous Internal Fixation versus Open Reduction Internal Fixation for Low Energy Tibia Plateau Fractures

**DOI:** 10.1038/s41598-018-32201-y

**Published:** 2018-09-19

**Authors:** Yiyang Wang, Jianping Wang, Jun Tang, Feiya Zhou, Lei Yang, Jianbin Wu

**Affiliations:** The Second Affiliated Hospital and Yuying Children’s Hospital of Wenzhou Medical University, Zhejiang Province, China

## Abstract

The purpose of our study was to compare the curative effect of two surgical methods for Schatzker type I to III tibia plateau fractures, arthroscopy assisted reduction percutaneous internal fixation (ARIF) and open reduction internal fixation (ORIF), with the intent of evaluating the quality of evidence to assist treatment selection. Searches of PubMed, Cochrane and China National Knowledge Infrastructure (CNKI) databases were performed to identify randomized controlled trials (RCTs) and quasi-RCTs comparing ARIF and ORIF regarding the following outcomes: functional outcomes, perioperative complications and post-traumatic osteoarthritis. Odds ratios (OR) and weighted mean differences (MDs) were pooled using either a fixed-effects model or random-effects model, depending on the heterogeneity of the trials included in the analysis. 19 RCTs and one quasi-RCT provided the data from 1272 patients. ARIF was associated with better functional outcomes, a lower risk of perioperative complications, and lower risk of post-traumatic osteoarthritis. After consideration of the quality of evidence of the included studies, the advantages provided by ARIF are not substantive over ORIF for the treatment of Schatzker type I to III tibia plateau fractures, except reducing the risk of perioperative complications.

## Introduction

Tibia plateau fractures are common intra-articular injuries sustained in the lower extremities^1^. There are two principles in the treatment of tibia plateau fractures, one is anatomical reduction of the articular surface and reconstruction of the mechanical axis of the lower limb; the other is to reconstruct the stability of the injured knee joint^2,3^. According to Schatzker classification system, type I to III fractures are low energy injuries, sustained in lateral tibia plateau^4–6^. Traditionally, displaced tibia plateau fractures are treated with open reduction internal fixation (ORIF), even in Schatzker type I to III fractures^7,8^. Inevitably, infections, hematoma formation, surgical wound dehiscence and surgical wound edge necrosis are frequent complications, due to extensive soft tissue dissection during ORIF^2,9–11^. The anterior-lateral approach is the most widely used surgical approach when treating tibia plateau fractures surgically, especially in treating fractures located in the lateral tibia plateau^12,13^. The iliotibial band should be released from the Gerdy’s tubercle, and should be incised a little above the knee joint line^2,11^. Even more, arthrotomy of the knee joint should be performed, and the lateral meniscus should be released from the lateral tibia plateau, in order to assess fracture reduction directly^2,11^. The function of the knee joints may be affected by scar formation in the soft tissue aforementioned. Arthroscopy assisted reduction percutaneous internal fixation (ARIF) is emerging recently as an alternative treatment method in treating lower energy tibia plateau fractures^14–18^. The main advantage of this method relied on the minimally invasive nature during operation without violating the structures aforementioned^14,15,17,18^. There is controversy over the effectiveness of the two treatment methods, ORIF and ARIF concerning treatment of lower energy tibia plateau fractures. Accordingly, we performed this meta-analysis to compare the treatment effect of arthroscopy assisted reduction percutaneous internal fixation, in treating Schatzker type I to III tibia plateau fractures, with that of open reduction internal fixation.

## Materials and Methods

### Search strategy

We searched PubMed, Cochrane and China National Knowledge Infrastructure (CNKI) databases from their inception to September 2016, without search filters, using the following MeSH (Medical Subject Heading) terms and text words: tibia fractures, knee injuries, knee joint/injuries, tibial fractures, tibia plateau fractures, tibial plateau fractures, proximal tibia fractures, proximal tibial fractures, proximal metaphyseal tibia fractures, proximal metaphyseal tibial fractures, proximal epiphyseal tibia fractures, proximal epiphyseal tibial fractures, fracture fixation, fracture osteosynthesis, bone nails, bone plates, external fixation, internal fixation, plates, extramedullary fixation, osteosynthesis, intramedullary nails, external fixators, circular fixators, hybrid external fixators. (Supplemental File 1) We also searched the reference lists of the relevant studies identified to supplement our literature search.

### Inclusion criteria/exclusion criteria

Only RCTs and quasi-RCTs were enrolled in our study, with non-randomized trials excluded. All the RCTs and quasi-RCTs comparing ORIF to ARIF for low energy Schatzker type I to III tibia plateau fractures were eligible, with open fractures excluded, the duration from injuries to operation should not beyond 3 weeks.

### Outcomes of Interest

We included the following outcomes of interest in our analysis: the functional outcomes measured by validated scales; perioperative complications (infection, wound dehiscence, hematoma formation, surgical wound edge necrosis, nerve injuries, vascular injuries, compartment syndromes, and deep vein thrombosis); post-traumatic osteoarthritis.

### Study Selection and Data Extraction

Two of our reviewers assessed the eligibility of the identified trials independently. We developed a data extraction sheet based on the Cochrane Consumers and Communication Review Group’s data extraction template^19^. Two reviewers collected data independently; disagreements were resolved by discussion with a senior author if necessary. We extracted the following information from the included RCTs or quasi-RCTs: research method; the inclusion and exclusion criteria of the included trials; characteristics of trial participants; fracture classification; interventions characteristics, such as the method of reduction and fixation; post-operative outcomes of interest; and risk of bias. We also attempted to contact the primary author through email to seek clarification, if information was missing.

### Quality Assessment

Two review authors used the domain-based evaluation described in the Cochrane Handbook for Systematic Reviews of Interventions to evaluate the risk of bias independently^20^. We assessed the following five domains: random sequence generation; allocation concealment; methods of blinding, including blinding of participants, research personnel and outcome assessors; incomplete outcome data; and selective outcome reporting. Each of these criteria was assessed as ‘low risk of bias’ or ‘high risk of bias’. When there was lack of information or uncertainty over the potential for bias, then they were assessed as ‘unclear risk of bias’. The GRADE approach was used to quantify the quality of the evidence^21^, when disagreements between the review authors regarding the risk of bias for the identified domains appeared, they were resolved by consensus.

### Statistical analysis

We used the Review Manager software (RevMan Version 5.3; The Nordic Cochrane Center, Denmark) to conduct the meta-analyses. Additionally, we used the Stata software (StataSE 12.0; StataCorp LP, USA) to perform the tests for funnel plot asymmetry. Dichotomous outcomes were calculated using Odds Ratios (ORs), together with 95% confidence intervals (CIs). Mean differences (MDs), with corresponding 95% CIs, were used to express continuous outcomes. When the same outcome was measured using different scales, or the same outcome was calculated with either dichotomous and continuous data, the standardized mean difference (SMD) was calculated. We used chi-squared analysis to test the heterogeneity across the included trials, and we used the I^2^ statistic to assess the impact of identified heterogeneity on the analysis. If the I^2^ was greater than 50%, heterogeneity was defined as substantial. Estimates of the pooled data were evaluated using a random-effect model, when the substantial heterogeneity between trials included in an analysis was identified; otherwise, a fixed-effect model was chosen. Both the Begg and Egger tests were used to assess the funnel plot asymmetry.

## Results

### Search Results

**Figure 1:**
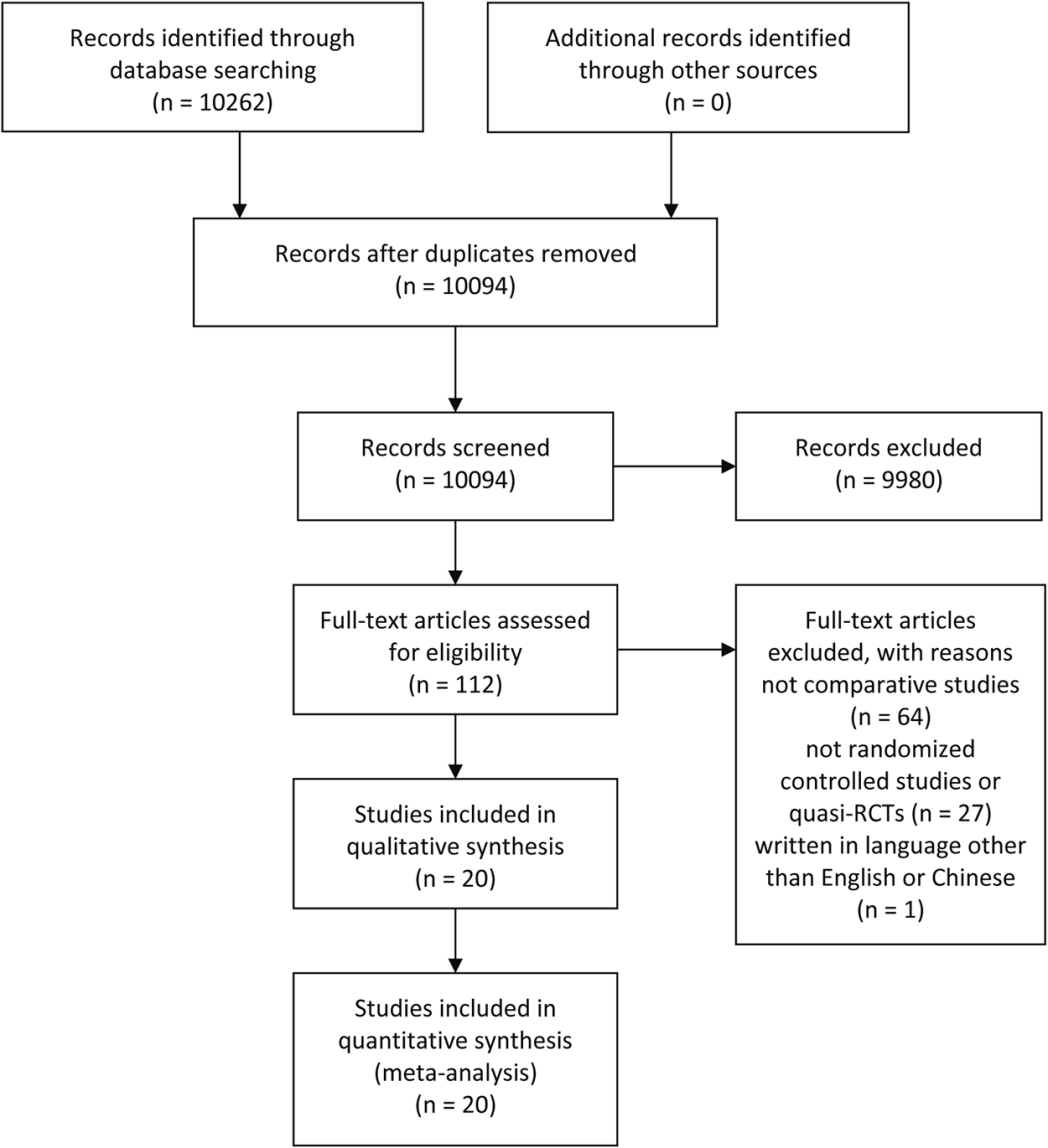
Flow Diagram of Search.

Figure 1 presents the details of the literature search strategy and exclusion criteria in the flow diagram. We identified and screened a total of 10261 titles and abstracts, eventually 20 trials satisfied our eligibility criteria^22–41^. 19 of the included trials were RCTs^23–41^, with one quasi-RCT^22^, comparing ORIF and ARIF in the treatment of closed Schatzker type I to III tibia plateau fractures in adults.

### Quality Assessment

**Figure 2:**
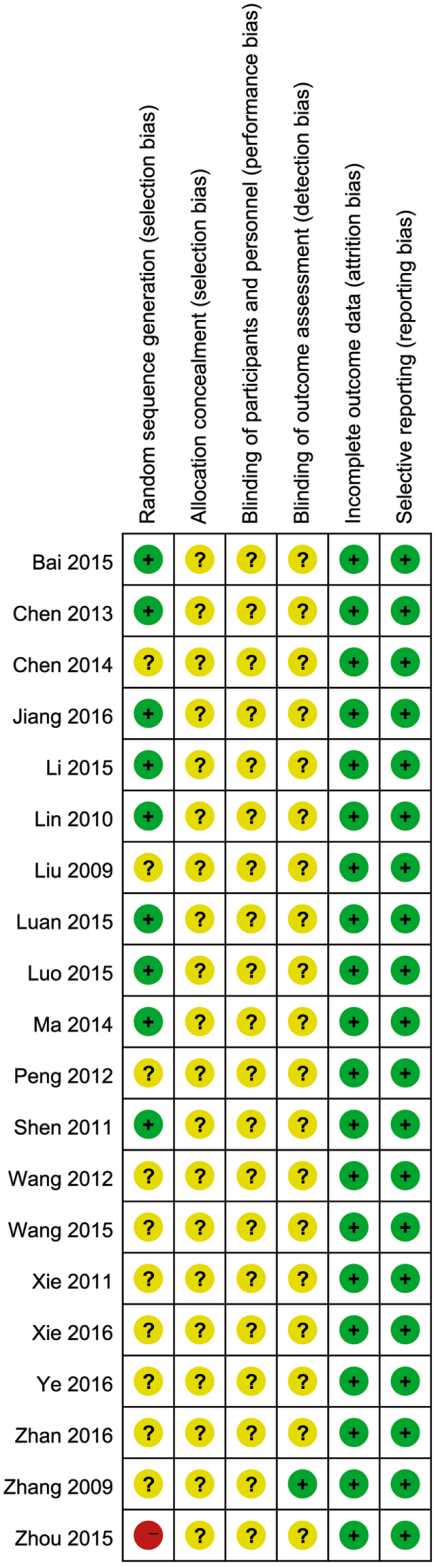
Summary of Risk Bias Assessment. Note: Reviewers’ assessment of each risk of bias item; “+”, low risk of bias; “?”, unclear risk of bias; and “−”, high risk of bias.

**Figure 3:**
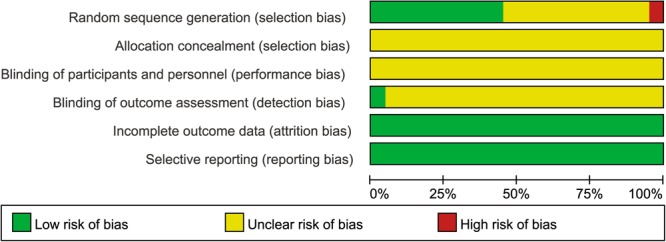
Risk of Bias Graph. Note: Reviewers’ assessment of each risk bias item, presented as a percentage across all included RCTs.

All the included studies had serious methodological flaws that put them at either unclear or high risk of bias for at least three domains (see Figs 2 and 3). Adequate randomization method was described in 9 RCTs^23,25–27,29,33,35,36,40^, table of random number was used to generate random sequence. The other 10 RCTs did not describe the method of generation of randomization sequence^24,28,30–32,34,37–39,41^. The one quasi-RCT stated that the random sequence was based on admission number^22^. None of the included trials described the method of allocation concealment. Only one included trial confirmed the blinding of outcome assessors^32^, while the other trials did not inform status of blinding.

### Descriptive Characteristics

All the included studies were single-center trials conducted in China, and all were reported in Chinese. There were only two intervention groups in the all included trials. Open tibia plateau fractures were not included in all 20 trials. Together, the included trials enrolled a total of 1272 patients, providing 1272 fractures, in our analysis. All of the included trials enrolled low energy lateral tibia plateau fractures, with one trial only including Schatzker type III fractures^36^, one trial including Schatzker type I and II fractures^39^, 9 trials including Schatzker type II and III fractures^22,25,26,28,29,31–34^, 9 including Schatzker type I to III fractures^23,24,27,30,35,37,38,40,41^. All the included trials stated that ORIF was performed through an anterior-lateral surgical approach, arthrotomy was performed from lateral knee compartments, with lateral meniscus being elevated, split fractures of the lateral tibia plateau were reduced by reduction clamp, depressed fractures were reduced by impacting the subchondral bone through the fracture gap or the created bony window, fixation were achieved with plates or screws; ARIF were performed through standard arthroscopic portals, reduction of the split fractures were achieved by reduction clamps, depressed fractures were elevated through the cortical window or the fracture gap using impactors under control of arthroscopy, fixation are achieved with plates or screws. The descriptive characteristics of the included studies are listed in Table 1.

**Table 1:** Descriptive Characteristics of Included Trials.

Study	Year	Sample Size (ARIF)	Sample Size (ORIF)	Gender (M/F) (ARIF)	Gender (M/F) (ORIF)	Age (ARIF)	Age (ORIF)	Follow-up Duration (Mo) (ARIF)	Follow-up Duration (Mo) (ORIF)	Schatzker classification	Functional Scale
Zhou	2015	32	32	19/13	20/12	45.3 ± 6.5	42.5 ± 7.2	13.50 ± 1.07	13.80 ± 1.14	II, III	HSS
Bai	2015	64	64	N/A	N/A	N/A	N/A	12	12	I, II, III	Rasmussen
Chen	2014	36	21	N/A	N/A	N/A	N/A	14	14	I, II, III	HSS
Li	2015	34	33	20/14	18/15	47.3 ± 14.9	45.9 ± 15.2	15.8 ± 3.7	15.8 ± 3.7	II, III	Rasmussen
Lin	2010	20	30	15/5	21/9	44.7 ± 1.31	45.1 ± 13.5	10.60 ± 3.44	10.80 ± 3.09	II, III	HSS
Luan	2015	23	22	16/7	14/8	47.4 ± 12.7	48.5 ± 11.8	9.30 ± 1.5	9.50 ± 1.60	I, II, III	Lysholm
Peng	2012	34	34	*	*	*	*	13	13	II, III	HSS
Shen	2011	38	20	26/12	14/6	36.1	36.8	13.1	13.4	II, III	HSS
Wang	2012	19	19	12.7	13.6	49	51	6–16	6–16	I, II, III	Lysholm
Xie	2010	5	5	**	**	**	**	25	25	II, III	Lysholm
Zhang	2009	20	20	12.8	11.9	42.5	43.5	12	12	II, III	Lysholm
Luo	2015	54	54	29/25	28/26	34.3 ± 0.32	36.5 ± 0.42	N/A	N/A	II, III	Rasmussen
Zhan	2016	34	34	19/15	20/14	39.4 ± 3.8	40.3 ± 4.2	N/A	N/A	II, III	Lysholm
Ma	2014	48	48	27/21	26/22	34.2 ± 8.5	33.8 ± 7.3	12	12	I, II, III	HSS
Jiang	2016	40	40	28/12	29/11	58.6 ± 7.1	58.1 ± 7.3	N/A	N/A	III	Rasmussen
Wang	2015	20	20	13/7	12/8	34.8 ± 5.1	35.3 ± 4.6	N/A	N/A	I, II, III	HSS
Xie	2016	33	33	17/16	18/15	40.2 ± 2.2	41.5 ± 2.2	N/A	N/A	I, II, III	Rasmussen
Ye	2016	36	36	***	***	***	***	N/A	N/A	I, II	Rasmussen
Chen	2013	44	44	22/22	23/21	45.1 ± 12.8	45.9 ± 12.7	N/A	N/A	I, II, III	Rasmussen
Liu	2009	15	14	9/6	8/6	34.2 ± 6.0	35.4 ± 3.6	N/A	N/A	I, II, III	Rasmussen

Note: ARIF, arthroscopy assisted reduction percutaneous internal fixation; ORIF, open reduction internal fixation; M/F, male/female; Mo, month; HSS, Hospital for Special Surgery knee-rating score; Rasmussen, Rasmussen clinical assessment score; Lysholm, Lysholm score; N/A, Not Available; *a general M/F ratio of 50/18, a mean age was 36.4 ± 9.0 years; **a general M/F ratio of 5/5, a mean age was 39.5 years; ***a general M/F ratio of 39/33, a mean age was 42.4 ± 17.5 years.

### Effects of Interventions

#### Functional Outcome

**Figure 4:**
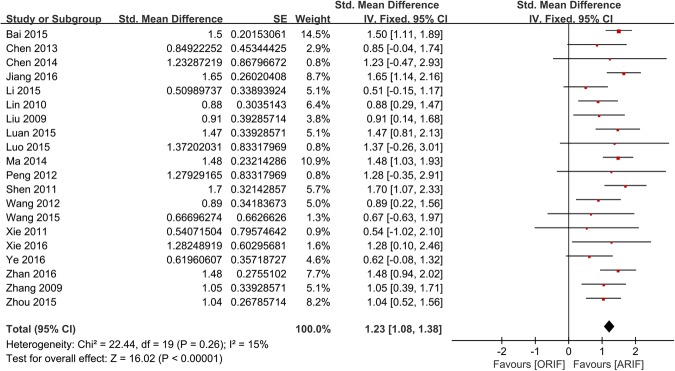
Forest Plot of SMDs and Associated 95% Confidence Intervals for Functional Outcomes.

3 different validated scales were used across the included trials to measure the patients’ functional scores: 7 trials used the Hospital for Special Surgery knee-rating score (HSS);^22,24,26,28,29,35,37^ 5 trials the Lysholm score^27,30–32,34^; 8 the Rasmussen clinical assessment score^23,25,33,36,38–41^. Pooled data, shown in Fig. 4, indicated statistically significant better post-operative functional outcomes for patients treated with ARIF, compared to ORIF (SMD = 1.23, 95% CI, 1.08–1.38; *p* < 0.00001).

#### Perioperative Complications

**Figure 5:**
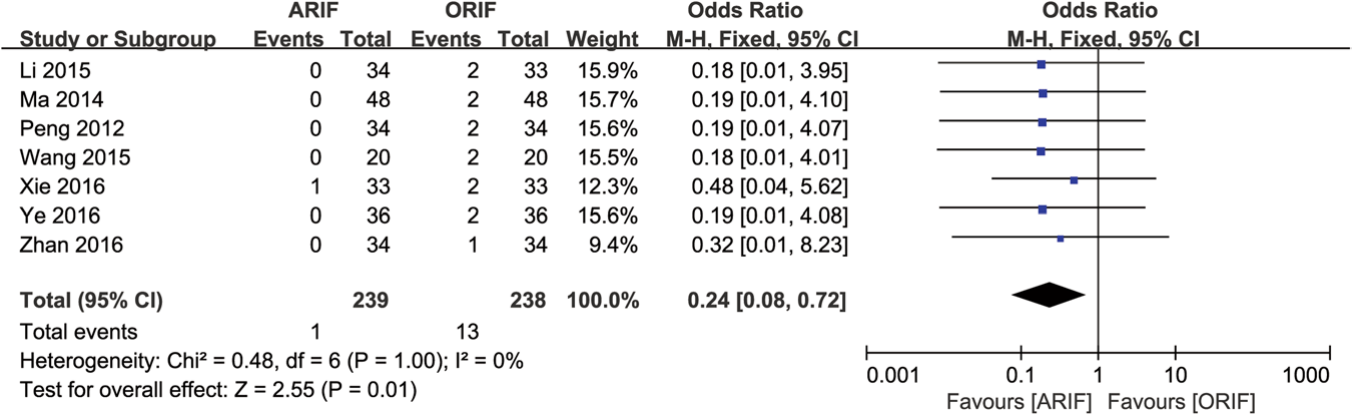
Forest Plot of OR, and Associated Confidence Intervals, for perioperative complications.

Although perioperative complications were reported in 14 trials^22–26,28,29,31,34,35,37–40^, there were 4 trials that reported no perioperative complications in either ARIF and ORIF group, which could not be estimated with Odds Ratio, so we included the other 10 trails in the analysis, with a calculated OR of 0.29 (95% CI, 0.15–0.55; *p* = 0.0002; Fig. 5). The perioperative complications in ORIF group included 22 infections, 4 surgical wound dehiscence, 4 surgical wound necrosis, 4 deep vein thrombosis, and 2 compartment syndromes. The perioperative complication in ARIF group included 7 infections, one wound dehiscence, one wound necrosis, and three deep vein thrombosis. The authors didn’t describe the severity, treatment and prognosis of these perioperative complications.

#### Post-traumatic Osteoarthritis

**Figure 6:**
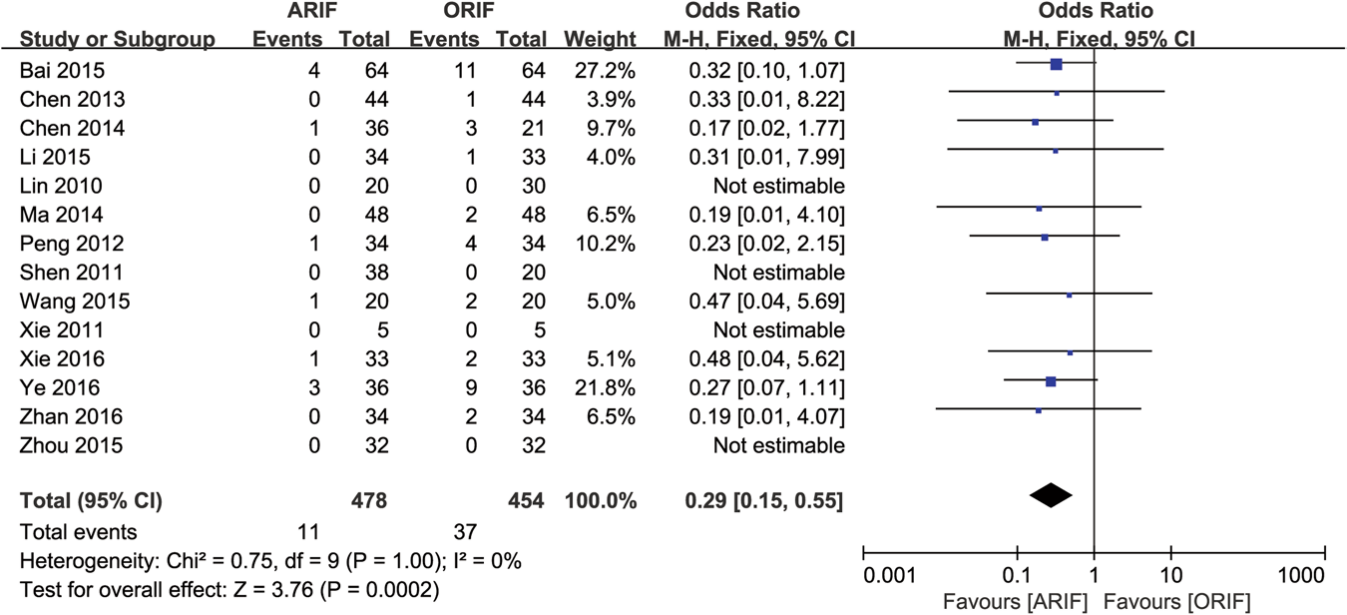
Forest Plot of OR, and Associated Confidence Intervals, for post-traumatic osteoarthritis.

7 trials reported the outcome of post-traumatic osteoarthritis^25,28,34,35,37–39^, with a calculated OR of 0.24 (95% CI, 0.08–0.72; *p* = 0.01; Fig. 6).

#### Publication Bias

**Figure 7:**
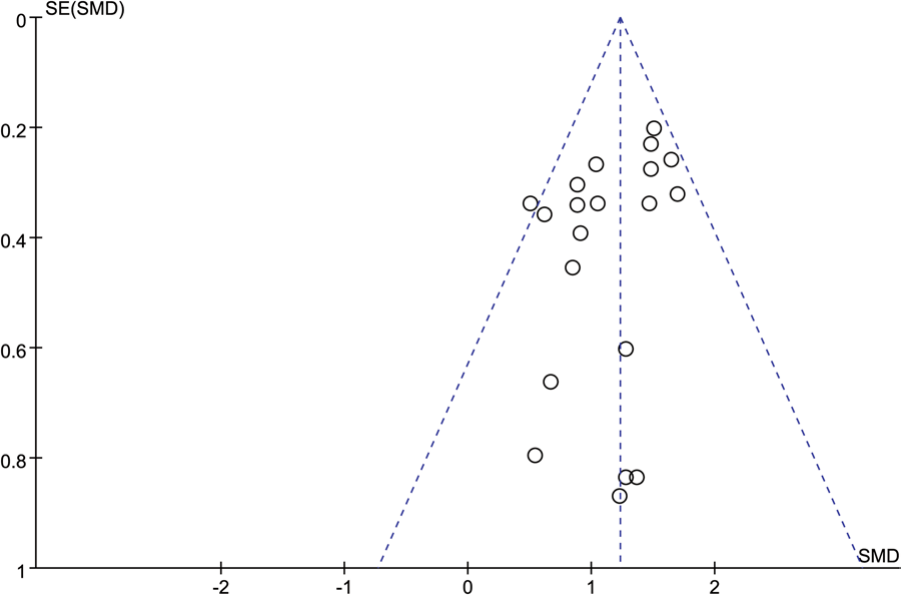
Funnel Plot of SMD and SE, for functional outcomes.

**Figure 8:**
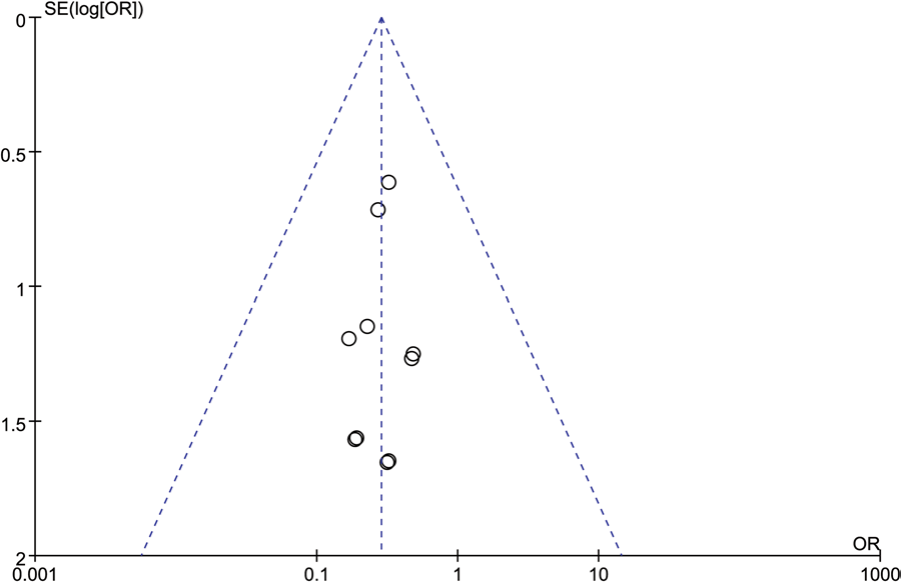
Funnel Plot of OR and Associated Confidence Intervals, for perioperative complications.

**Figure 9:**
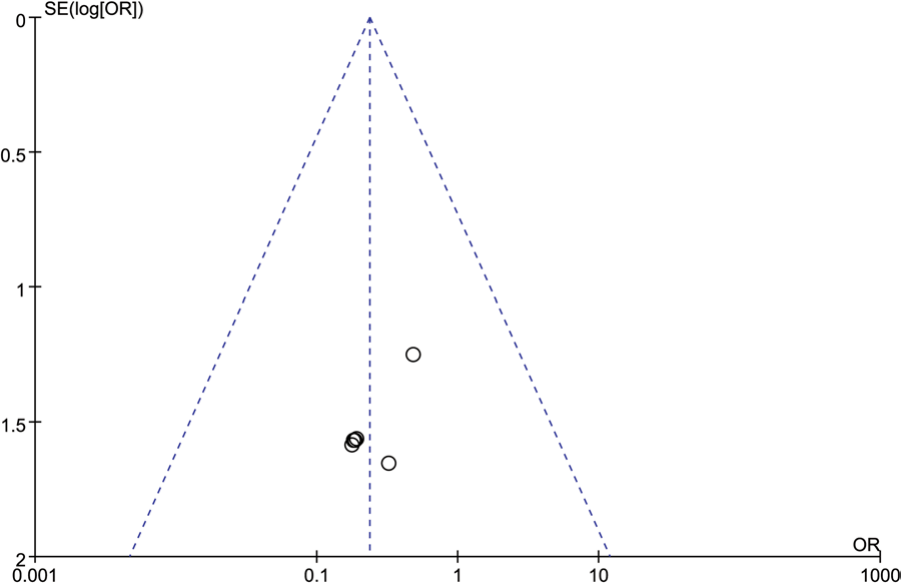
Funnel Plot of OR and Associated Confidence Intervals, for post-traumatic osteoarthritis.

We conducted the assessments of publication bias for the factors of the functional outcomes, perioperative complications, and post-traumatic osteoarthritis. Both Egger’s test and Begg’s test did not identify any potential publication bias in the functional outcomes (Egger’s test, P = 0.104; Begg’s test, *p* = 0.229), and perioperative complications (Egger’s test, *p* = 0.662; Begg’s test, *p* = 0.721). Potential publication bias was identified in post-traumatic osteoarthritis in Egger’s test (*p* = 0.032), but not in Begg’s test (*p* = 0.133). The funnel plots of these three outcomes of interest are illustrated in Figs 7–9.

## Discussion

Tibia plateau fractures are frequent injuries sustained in the lower extremities^1^. The anterior-lateral surgical approach is often used to reduce and internally fixate tibia plateau fractures, especially in lateral tibia plateau injuries^12,13^. Traditionally, the purpose of arthrotomy of the lateral knee joint compartment and elevation of meniscus from tibia plateau in Schatzker I to III fractures is to assess the effect of fractures reduction directly, decompression of the articulate surface is completed from the fracture gap or bony window in metaphyseal area^42–44^. With the development of arthroscopic technique, the reduction assessment could be achieved without arthrotomy, the fracture fixation could be completed in minimally invasive fashion^45–47^. In recent years, there’s trend to treat low energy tibia plateau fractures using ARIF technique in China, but there are no clear guidelines as to the superiority between ARIF and ORIF in the treatment of Schaztker I to III fractures. The purpose of our meta-analysis is to critically compare the functional outcomes and complications, between ARIF and ORIF in treating low energy tibia plateau fractures.

Our meta-analysis indicates that the difference in functional outcomes is statistically significant between the two groups: ARIF is superior than ORIF. All the function scales used in the included trials are validated in the literature^48–50^. We deduce that the integrity of the lateral knee capsule and the avoidance of meniscus detachment preserve the maximum function of the affected knees. In accordance with other minimally invasive procedures in treating fractures other than tibia plateau fractures, risk of perioperative complications in ARIF group is statistically significantly lower than ORIF group in our meta-analysis^9,51^. Surgical site infections are the most common perioperative complications. We thought the there would be a difference between the aggressive nature of ORIF and the minimally invasive nature of ARIF. Although our included trials didn’t describe the management and prognoses of these complications, we suppose the decrease of perioperative complications could at least shorten the length of hospital stay, reduce the patient’s suffering and the practitioners’ workload. The post-traumatic osteoarthritis of the knee joints is a severe complication in the long term; it is complex and challenging to manage^52,53^. Our meta-analysis indicated that there is a statistically significant lower risk of post-traumatic osteoarthritis in ARIF group. Although there were 7 trials that reported the outcome of post-traumatic osteoarthritis, only 2 trials reported the follow-up duration, none of the follow-up duration exceeded 24 months postoperatively, whilst none of the other 5 trials stated the length of the follow-up period. None of the included trials described the diagnostic criteria of post-traumatic osteoarthritis.

None of the included studies gave information about allocation concealment, and almost none of the included trials described the method of blinding of participants, practitioners and outcome assessment. Because of the surgical intervention nature of our included trials, we suppose it is hard to execute allocation concealment and blinding. Also taking into account the fact that only 9 of the included trials reported methods of random sequence generation, we conclude that the quality of the data for the functional outcomes to be low. Bearing in mind the aforementioned reasons, but due to the large effect, we deduced the quality of the data for the outcome of perioperative complications to be moderate. Due to the limited information of diagnostic criteria and follow-up duration, combined with the potential for publication bias, we reasoned the quality of the data for incidences of post traumatic osteoarthritis to be very low.

Our meta-analysis searched multiple data bases, using numerous MeSH terms and text words, without search filters. The search strategy and research protocol strictly adhered to the PRISMA statement for reporting systematic reviews and meta-analysis^54^. (Supplemental File 2) The detailed procedure in the two interventions, namely ARIF and ORIF, are highly coincident in the included trials. Still, there are several limitations in our meta-analysis. First, all the included trials were conducted in China, and all were written in Chinese. Second, all the included trials were small, single center studies. Third, although we only included Schaztker I to III fractures, the concomitant injuries such as meniscus injuries, collateral ligaments injuries, and cruciate ligaments injuries were different between the included studies. Fourth, because of language barriers, we excluded studies written in languages other than English and Chinese. We acknowledge that failure to include studies in other languages will result in missing data. Fifth, treating tibial plateau fractures through ARIF is much more technically demanding than ORIF, there will be a long learning curve confronted by the treating surgeons, the treating surgeons’ experience should be heterogeneous in the included studies. But the focus of our study is Schaztker I to III tibia plateau fractures, which are low energy injuries and easy to reduce and fix, the operative procedures are much more programmatic compared with high energy tibia plateau fractures.

In summary, out study is the first meta-analysis to compare functional outcomes and complications between ARIF and ORIF for the treatment of low energy tibia plateau fractures. Based on the evidence evaluated, ARIF provided no substantive advantage over ORIF in treating Schaztker I to III tibia plateau fractures, except in reducing the risk of perioperative complications. In the future, we need multicenter RCTs, with high methodological quality and long follow-up duration to inform the differences in functional outcomes and risk of post traumatic osteoarthritis between these two treatment methods.

## Electronic supplementary material

Supplemental file 1 (search strategy)

Supplemental file 2 (PRISMA Checklist)
