# Supplementary material for: Arthroscopy Assisted Reduction Percutaneous Internal Fixation versus Open Reduction Internal Fixation for Low Energy Tibia Plateau Fractures
Source: Sci Rep. 2018 Sep 19;8:14068. doi: 10.1038/s41598-018-32201-y (PMC6145938; doi:10.1038/s41598-018-32201-y)
Supplement: Supplementary file 1 — Supplemental file 1 (search strategy) [file 41598_2018_32201_MOESM1_ESM.pdf]

Arthroscopy Assisted Reduction Percutaneous Internal Fixation versus Open Reduction Internal Fixation for Low Energy Tibia Plateau Fractures

Yiyang Wang[1], Jianping Wang[1], Jun Tang[1], Feiya Zhou[1], Lei Yang[1], Jianbin Wu[1]*

[1]The Second Affiliated Hospital and Yuying Children's Hospital of Wenzhou Medical University, Zhejiang Province, China

Yiyang Wang, e-male: fey10000@163.com

Jianping Wang, e-male: wjp692821@163.com

Jun Tang, e-male: 13868656511@139.com

Feiya Zhou, e-male: feiyazhou@yeah.net

Lei Yang, e-male: wyey_yl@hotmail.com

*Jianbin Wu, corresponding author, e-male: wjb@wzhealth.com

Search strategy (PubMed)

#1 Tibial Fractures [MeSH Terms]

#2 Knee Injuries [MeSH Terms]

#3 knee joints/injuries [MeSH Terms]

#4 #1 or #2 or #3

#5 "tibia fracture" or "tibia fractures" or “fracture of tibia” or “fractures of tibia” or "tibial fracture" or "tibial fractures" or "tibia plateau fracture" or "tibia plateau fractures" or "tibial plateau fracture" or "tibial plateau fractures" or "proximal tibia fracture" or "proximal tibia fractures" or "proximal tibial fracture" or "proximal tibial fractures" or "fracture of tibia plateau" or "fractures of tibia plateau" or "fracture of tibial plateau" or "fractures of tibial plateau" or "fracture of proximal tibia" or "fractures of proximal tibia" or “knee injury” or “knee injuries” or “proximal metaphyseal tibia fracture” or “proximal metaphyseal tibia fractures” or “proximal metaphyseal tibial fracture” or “proximal metaphyseal tibial fractures” or “proximal epiphyseal tibia fracture” or “proximal epiphyseal tibia fractures” or “proximal epiphyseal tibial fracture” or “proximal epiphyseal tibial fractures”

#6 #4 or #5

#7 “tibial shaft fracture” or “tibial shaft fractures” or “tibia shaft fracture” or “tibia shaft fractures” or “tibial diaphyseal fracture” or “tibial diaphyseal fracture” or “tibia diaphyseal fracture” or “tibia diaphyseal fractures” or “tibial diaphysial fracture” or “tibial diaphysial fracture” or “tibia diaphysial fracture” or “tibia

diaphysial fractures” or “tibial diaphysis fracture” or “tibial diaphysis fracture” or “tibia diaphysis fracture” or “tibia diaphysis fractures” or “distal tibial fracture” or “distal tibial fractures” or “distal tibia fracture” or “distal tibia fractures” or “pilon fracture” or “pilon fractures”

#8 #6 not #7

#9 fracture fixation, internal [MeSH Terms]

#10 Fracture Fixation, Intramedullary [MeSH Terms]

#11 fracture fixation [MeSH Terms]

#12 fracture osteosynthesis [MeSH Terms]

#13 bone nails [MeSH Terms]

#14 bone plates [MeSH Terms]

#15 External Fixators [MeSH Terms]

#16 #9 or #10 or #11 or #12 or #13 or #14 or #15

#17 "fracture fixation" or "fractures fixation" or "internal fixation" or "plates" or "plate" or "bone plates" or "bone plate" or "extramedullary fixation" or "osteosynthesis" or “intramedullary nail" or "intramedullary nails" or "nail" or "nails" or “external fixator” or “external fixators” or “circular fixator” or “circular fixators” or “hybrid external fixator” or “hybrid external fixators”

#18 #16 or #17

#19 #8 and #18
